# Epidemiologic application of verbal autopsy to investigate the high occurrence of cancer along Huai River Basin, China

**DOI:** 10.1186/1478-7954-9-37

**Published:** 2011-08-04

**Authors:** Xia Wan, Maigeng Zhou, Zhuang Tao, Ding Ding, Gonghuan Yang

**Affiliations:** 1 Institute of Basic Medical Sciences of Chinese Academy of Medical Sciences/School of Basic Medicine of Peking Union Medical College, Beijing, China; 2 Chinese Center for Disease Control and Prevention, Beijing, China; 3 Graduate School of Public Health, San Diego State University, San Diego, California, USA; 4 School of Medicine, University of California, San Diego, San Diego, La Jolla, California, USA

**Keywords:** Verbal autopsy, cancer, mortality rate, prevalence rate, water pollution

## Abstract

**Background:**

In 2004, the media repeatedly reported water pollution and "cancer villages" along the Huai River in China. Due to the lack of death records for more than 30 years, a retrospective survey of causes of death using verbal autopsy was carried out to investigate cancer rates in this area.

**Methods:**

An epidemiologic study was designed to compare numbers of deaths and causes of death between the study areas with water pollution and the control areas without water pollution in S County and Y District in 2005. The study areas were selected based on the distribution of the Huai River and its tributaries. Verbal autopsy was used to assist cause of death (COD) diagnoses and to verify mortality rates. The standard mortality rates (SMRs) of cancer in the study area were compared with those in the control areas. In order to verify the difference between mortality rates due to cancers in the study and the control areas, patients who reported having cancer in the survey received a second diagnosis by national and provincial oncologists with pathological and laboratory examinations. Comparisons were made to determine if differential cancer prevalence rates in the study and control areas were similar to the difference in mortality due to cancer in these study and control areas. Mortality rates of cancers in study and control areas were also compared with national statistics for the rural population of China.

**Results:**

Over five years, 3,301 deaths were identified, including 1,158 cancer deaths. The annual average SMRs of cancer in the study areas of S County and Y District were 277.8/100,000 and 223.6/100,000, respectively, which is three to four times higher than those in the control areas. In addition, a total of 626 cases of cancer in the study and control areas were confirmed. The prevalence rates of cancer were 545/100,000 and 128.1/100,000 per year in the study and control areas in S County, respectively, and 440.9/100,000 and 200/100,000 per year in the study and control areas in Y District, respectively. The mortality and prevalence rates of digestive cancers were higher in the study areas than the control areas. In 2000, the SMR for cancer in rural areas nationwide was 120.9/100,000, and in study areas in S County and Y District, the excess rates of deaths were 184/100,000 and 138.8/100,000, respectively.

**Conclusions:**

The death rates of digestive cancers were much higher in the study areas of S County and Y District. The patterns for between-area differences in prevalence and mortality rates of cancer were similar. Verbal autopsy is shown to be a useful tool in retrospective mortality surveys in low-resource areas with limited access to health care.

## Background

In 2004, China Central Television and other media outlets reported water pollution and "cancer villages" [1] along the Huai River, which caused concern in the public and the government. An important question to be answered is whether cancer prevalence and mortality rates are significantly higher in these villages. Normally, an investigation of this of type would depend on a vital registration system with accurate data, especially on COD. However, there has been no routine vital registration system on COD in these impoverished areas, and the only retrospective survey of COD was conducted from 1973 to 1975 [2]. In addition, there were no population-based reports on COD in the following years. To answer the above research question, a retrospective survey of death causes was conducted using verbal autopsy (VA).

VA is an interview carried out with family members and/or caregivers of the deceased, using a structured questionnaire to elicit signs and symptoms and other pertinent information that can be used to assign a probable underlying cause of death [3]. The VA procedure was validated for adult deaths in China in 2005 [4], and the sensitivity and specificity for cancers exceeded 85% and 95%, respectively. Moreover, VA has been used to evaluate the quality of the urban [5] and rural [6] COD reporting system of China's Disease Surveillance Points (DSP) System, demonstrating the feasibility of using this tool in practice.

In the current study, we use the validated VA to determine whether a high prevalence of cancer exists along the Huai River basin. Findings will influence further research on the relationship between water pollution and cancers.

## Methods

Based on the distribution of the Huai River and its tributaries, an epidemiologic study was designed to compare death rates and COD between the study areas with water pollution and the control areas without water pollution. VA procedure was used to assist COD diagnosis and to verify the mortality rates. The investigation was conducted from August to December 2005. The standard mortality rates (SMRs) of cancers in the study areas were compared with those in the control areas. In order to verify the difference in cancer-caused mortality rates between the study and the control areas, a prevalence survey of cancers was used to verify the mortality rates by VA. Additionally, mortality rates of cancers in the study and control areas were compared with national statistics from China's rural population.

### Selection of study areas

**Figure 1:**
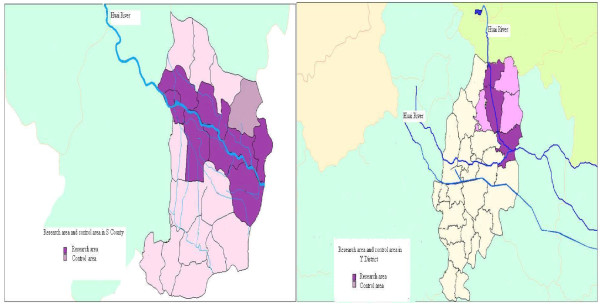
**Map of study and control areas in S County and Y District**.

The S County and Y District were selected for this study because they were reported to have high cancer rates, and they are adjacent to the Huai River. There was one study area and one control area in each county/district. In S County, the study area was irrigated by the main branch and stream of the Huai River, while the control area was up in the hills where the Huai did not pass through. In the Y District, the study area was selected within five kilometers from the main branch of the Huai River, and the control area was more than five kilometers away from the river (Figure 1). Based on the power and sample size calculations [7] (assuming the cancer mortality rates in the study and control areas are 0.03% and 0.01%, respectively), it was determined that 50,000 people from each study and control area should be investigated. Based on inclusion criteria and power and sample size calculations, 25 villages were selected as study areas and 19 were selected as control areas in S County, and 31 villages were selected as study areas and 32 as control areas in Y District.

### Death cause investigation with VA

The VA questionnaire was derived from standard questions [8], the operational characteristics of which have been previously assessed in China [4]. This questionnaire was used to ask a family member to report symptoms of the decedent, and the duration, diagnosis, and medication history during illness. In addition, the questionnaire also included open-ended questions to the respondent, including a narrative of events leading to death, diagnostic records by the hospital, medication records, etc. When available, hospital records, laboratory tests, and death certificates were photocopied and included in the review process. The questions asked family members varied by age, gender, and disease of the decedent. The total number of questions ranged from 66 to 82 and the interview time was about 30 to 40 minutes. Of all the questions, about 30 were related to symptoms caused by cancer (Additional file 1).

All of the deaths between July 1, 2002 and June 30, 2005 in the study and control areas were collected. Then, family members or main caregivers of the deceased above 5 years old were interviewed using the VA questionnaire. The interviews were conducted by local health workers who underwent a three-day training. Completed VA questionnaires were reviewed by an independent panel of local senior clinical experts who had completed a training course on how to use VA questions to diagnose specific cancers. Two experts independently identified the COD based on the information recorded on the form and in available records. Only one cause was assigned for each death. Each physician was unaware of the diagnosis assigned by the other physician. If the two diagnoses were discordant, the forms were reviewed by a third physician. A total of 11.7% of cases went to a third review. If the diagnoses of the three experts were not consistent with each other, the three experts were asked to discuss the case and to reach a consensus. If they could come to an agreement, the diagnosis would be accepted. Otherwise, the death was recorded as having an unidentified cause. Deaths with unclear diagnoses would be further diagnosed by a team of experts in Beijing. Deaths with an unspecified cause accounted for 6.6% of all cases. Finally, local encoding professionals carried out coding based on the 10^th ^revision of the International Classification of Diseases (ICD-10) for each VA questionnaire. Provincial and national ICD-10 coding experts randomly checked COD coding and corrected incorrect coding.

### Verification of COD diagnosis with prevalence surveys

In order to verify the difference in mortality caused by cancer between the study and control areas, a prevalence survey was used to determine if the difference in prevalence rates of cancers in the study and control areas were identical to the difference in mortality caused by cancer in the study and control areas.

First, lists of patients with cancer who were still alive during the survey period were enumerated by village doctors (Village doctors are primary caregivers in rural China who provide basic medical procedures or referrals to county-level medical facilities. There is usually one village doctor per village. Village doctors usually know each villager well and can easily contact villagers for medical purposes.) through contact with each individual in the villages of the study and control areas. Then, all of these patients were examined by provincial or national oncologists through review of their medical records. Based on the guidelines for cancer diagnosis, those patients with sufficient evidence were confirmed. This evidence usually involves pathological and laboratory examinations. Those with insufficient evidence were accepted for further diagnosis. In addition, a prevalence survey was conducted in patients. The prevalence survey included demographic characteristics, and other structured questions regarding disease-related symptoms and duration, hospital diagnosis level, diagnosis certificate, diagnosis record, and related laboratory examination.

### Data analysis

All statistical analyses were conducted using SAS 9.2 software (PUMC, China). Nationwide rural mortality rates came from the 2000 annual report of the DSP System [9]. The population census data from 2000 was used for the SMR [10]. The DSP System for COD from 1991 to 2000 covered 10 million people in 145 locations in all provinces by multiple-stratified random sampling. This nationally representative sample reflected regional population distributions, urban and rural areas, age and sex, and eastern, central, and western regions of the country.

Analytical procedures for age distribution in the study and control areas included descriptive statistics (mean, standard deviation) and student's t test. Mortality rates, prevalence rates, relative risk, 95% confidence interval (CI) for standardized mortality rate [11], and p-values using the cross product difference test method [11] of the study and control areas were calculated. The formula for 95% CI is:

Where p: standard mortality rate; u_α/2 _: limit value of U distribution; p_i_: age-specific mortality rate; q_i_: 1-p_i_; and W_i_: the proportion of age-specific standard population.

To calculate the excess mortality rates for cancers between the study and control areas and to compare those with the national statistics for the rural populations, the following formula was used: the excess mortality rates for cancer = ∑[(age-specific mortality rate for cancer in a certain area - age-specific mortality rate for cancer at the national level) × age-specific population in this area]/total population in this area

## Results

### Demographic characteristics of deaths

In total, 3,301 deaths in people above 5 years of age were identified, of which 1,158 cases were caused by cancers. Among the 1,158 people who died of cancer, 83.8% in the study areas and 83.5% in the control areas visited a county-level or above health facility. The differences in the age of the deceased between the study and control areas were not statistically significant (66.9 versus 68.1 years in S County, p = 0.17; 70.1 versus 71.6 years in Y District, p = 0.08). The cancer mortality rates in the study areas were higher than those in the control areas, regardless of age group (Table 1).

**Table 1:** Mortality rate (per 100,000) of main causes of death by age group in study and control areas in S County and Y District, July 2002-June 2005

		Male								Female							
	
		Study area				Control area				Study area				Control area			
	
	Disease	5-14	15-34	35-64	65+	5-14	15-34	35-64	65+	5-14	15-34	35-64	65+	5-14	15-34	35-64	65+
S County	Infectious diseases	0.0	3.7	23.7	140.3	0.0	0.0	19.9	77.0	0.0	0.0	16.9	83.7	0.0	0.0	3.3	50.1
	Neoplasms	38.7	11.0	386.3	2587.7	11.0	4.2	152.9	449.0	11.1	19.6	192.4	1548.8	0.0	4.4	82.1	238.0
	Blood and blood-forming organs	0.0	0.0	3.4	0.0	0.0	0.0	0.0	12.8	0.0	0.0	0.0	0.0	0.0	0.0	6.6	0.0
	Endocrine system	0.0	0.0	0.0	46.8	0.0	0.0	13.3	25.7	0.0	0.0	3.4	27.9	0.0	4.4	0.0	12.5
	Neuropsychiatric system	0.0	7.3	10.2	124.7	0.0	4.2	6.6	102.6	0.0	0.0	6.8	97.7	0.0	4.4	6.6	87.7
	Circulatory system	0.0	25.7	84.7	1091.2	0.0	0.0	76.4	769.7	0.0	0.0	47.3	906.9	0.0	0.0	42.7	927.0
	Respiratory system	0.0	3.7	40.7	592.4	0.0	0.0	16.6	500.3	0.0	0.0	6.8	669.7	0.0	4.4	16.4	400.9
	Digestive system	0.0	0.0	10.2	233.8	0.0	0.0	19.9	115.5	0.0	0.0	13.5	223.2	0.0	4.4	9.9	112.7
	Genitourinary system	0.0	0.0	0.0	46.8	0.0	0.0	3.3	12.8	0.0	0.0	10.1	14.0	0.0	0.0	0.0	12.5
	Maternity diseases	0.0	0.0	0.0	0.0	0.0	0.0	0.0	0.0	0.0	7.8	0.0	0.0	0.0	0.0	0.0	0.0
	Accident or injury	48.4	62.4	77.9	140.3	44.1	59.4	86.4	243.7	0.0	23.5	74.3	139.5	0.0	13.1	32.9	200.4
	Congenital malformation	0.0	3.7	3.4	0.0	0.0	0.0	0.0	0.0	11.1	0.0	0.0	0.0	0.0	0.0	0.0	0.0
	Other	0.0	7.3	6.8	109.1	11.0	4.2	10.0	192.4	0.0	0.0	0.0	251.2	0.0	0.0	9.9	488.5
	Total	87.1	124.8	647.2	5113.0	66.2	72.2	405.5	2501.6	22.3	51.0	371.4	3962.6	0.0	35.0	210.3	2530.4

Y District	Infectious diseases	0.0	4.0	28.8	67.8	0.0	0.0	13.9	13.1	0.0	0.0	11.4	75.0	0.0	0.0	0.0	39.7
	Neoplasms	17.7	12.1	392.8	1864.0	0.0	3.9	107.8	809.6	0.0	20.9	144.3	1114.0	0.0	8.1	68.4	304.5
	Blood and blood-forming organs	0.0	0.0	0.0	0.0	0.0	0.0	0.0	0.0	0.0	0.0	0.0	0.0	0.0	0.0	0.0	0.0
	Endocrine system	0.0	0.0	3.6	56.5	0.0	0.0	3.5	13.1	0.0	4.2	0.0	64.3	0.0	4.1	0.0	39.7
	Neuropsychiatric system	0.0	0.0	7.2	22.6	0.0	0.0	13.9	26.1	0.0	0.0	0.0	42.8	0.0	0.0	3.6	92.7
	Circulatory system	0.0	12.1	86.5	1107.1	0.0	11.7	90.4	1188.3	0.0	4.2	57.0	1135.4	0.0	0.0	46.8	1668.0
	Respiratory system	0.0	0.0	18.0	338.9	0.0	0.0	27.8	705.1	0.0	0.0	0.0	353.5	0.0	0.0	14.4	423.6
	Digestive system	0.0	4.0	25.2	169.5	0.0	3.9	24.3	195.9	0.0	0.0	15.2	192.8	0.0	0.0	18.0	198.6
	Genitourinary system	8.8	8.1	10.8	33.9	0.0	0.0	0.0	91.4	0.0	0.0	11.4	32.1	0.0	4.1	7.2	26.5
	Maternity diseases	0.0	0.0	0.0	0.0	0.0	0.0	0.0	0.0	0.0	0.0	0.0	0.0	0.0	8.1	3.6	0.0
	Accident or injury	44.1	44.3	50.5	101.7	21.6	46.8	66.1	248.1	9.5	12.5	30.4	96.4	12.1	12.2	36.0	304.5
	Congenital malformation	0.0	0.0	3.6	0.0	0.0	3.9	3.5	0.0	0.0	0.0	0.0	0.0	0.0	0.0	0.0	0.0
	Other	0.0	0.0	14.4	225.9	0.0	0.0	38.2	404.8	0.0	4.2	3.8	321.3	0.0	0.0	10.8	357.4
	Total	70.6	84.5	641.5	3987.8	21.6	70.2	389.4	3695.5	9.5	46.0	273.4	3427.6	12.1	36.6	208.7	3455.1

### Cause-specific mortality fractions for major causes of death

**Figure 2:**
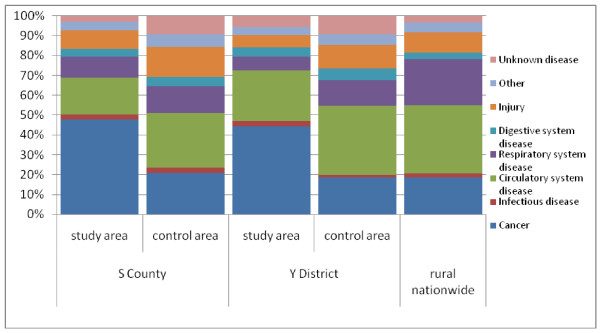
**Comparison of cause-specific mortality fractions for major causes of death in study and control areas in S County and Y District and rural areas nationwide**.

In the study areas of S County and Y District, the cancer mortality fractions were 48% and 44%, respectively, suggesting that cancer was the leading COD, while in the control areas those fractions were only about 20%, similar to the general rural population (Figure 2).

### Cancer mortality rates in the study areas

In the study areas in S County and Y District, the annual average SMR of cancer in people 5 years of age and above was 277.8/100,000 and 223.6/100,000, respectively, which is three to four times higher than the SMR in the control areas. The mortality rates of lung, stomach, esophageal, liver, and colorectal cancer in the study areas were two to six times higher than those in the control areas. The differences in cancer mortality between the study and control areas for different types of cancers were significant, except for colorectal cancer (Table 2).

**Table 2:** Comparison of mortality rates among people above 5 years of age in S County and Y District, July 2002-June 2005

	Disease	Number of deaths		Mortality rate (1/100,000)		SMR (1/100,000)* (95% CI)		Relative risk(RR)	p-value
				
		Study area	Control area	Study area	Control area	Study area	Control area		
S County	Total	972	617	671.4	439.0	586.1 (549.1, 623.1)	338.6 (311.6, 365.7)	1.7	0.000
	Total cancer	462	128	319.1	91.1	277.8 (252.4, 303.2)	72.1 (59.5, 84.7)	3.9	0.000
	Esophageal cancer	108	21	74.6	14.9	64.1 (52.0, 76.2)	11.8 (6.7, 16.8)	5.5	0.000
	Stomach cancer	83	14	57.3	10.0	49.4 (38.8, 60.0)	8.0 (3.8, 12.1)	6.2	0.000
	Liver cancer	84	25	58.0	17.8	50.2 (39.4, 60.9)	14.0 (8.5, 19.6)	3.6	0.000
	Colorectal cancer	14	5	9.7	3.6	8.4 (4.0, 12.8)	2.5 (0.3, 4.8)	3.3	0.018
	Lung cancer	107	36	73.9	25.6	63.6 (51.6, 75.7)	19.8 (13.3, 26.2)	3.2	0.000
Y District	Total	967	745	676.7	534.0	493.2 (461.4, 525.1)	415.7 (385.7, 445.8)	1.2	0.000
	Total cancer	429	139	300.2	99.6	223.6 (201.9, 245.3)	78.4 (65.3, 91.6)	2.9	0.000
	Esophageal cancer	51	22	35.7	15.8	25.7 (18.5, 33.0)	11.8 (6.8, 16.7)	2.2	0.002
	Stomach cancer	88	23	61.6	16.5	43.2 (33.9, 52.4)	12.9 (7.6, 18.3)	3.3	0.000
	Liver cancer	97	22	67.9	15.8	53.3 (42.4, 64.2)	12.6 (7.3, 17.9)	4.2	0.000
	Colorectal cancer	21	7	14.7	5.0	10.2 (5.7, 14.6)	4.0 (1.0, 7.0)	2.5	0.028
	Lung cancer	90	26	63.0	18.6	46.1 (36.3, 55.8)	14.6 (9.0, 20.3)	3.2	0.000

*: 2000 census data were used for the standard population

### Consistency in cancer mortality and prevalence rates

**Figure 3:**
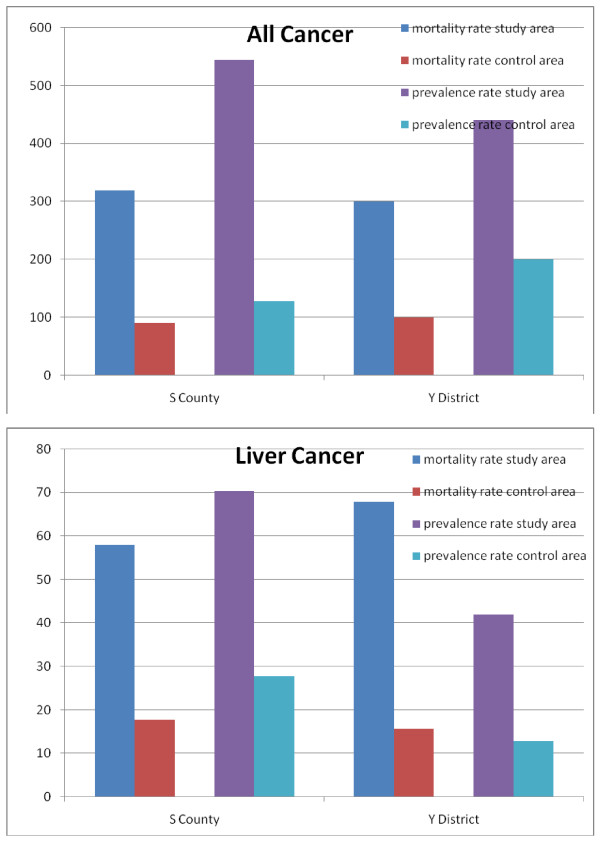
**Comparison of mortality and prevalence rates (1/100,000) for all types of cancer and liver cancer in S County and Y District, July 2002-June 2005**.

A total of 657 patients were reported to have cancer, of whom 37 had insufficient evidence for a cancer diagnosis. After further physical examination, 31 patients were excluded from having a diagnosis of cancer. In total, there were 626 cases of cancer in the study and control areas. In the study and control areas in S County, the prevalence rates were 545/100,000 and 128.1/100,000 per year, respectively. In the study and control areas in Y District, the rates were 440.9/100,000 and 200/100,000 per year, respectively. For all types of digestive cancer under investigation, the mortality and prevalence rates in the study areas were higher than those in the control areas (e.g., liver cancer [Figure 3]).

### Cancer mortality rates in study areas compared to the general rural population

In 2000, the SMR of cancer in people aged 5 years and above in rural populations nationwide was 120.9/100,000. Compared with this average mortality cancer rate, the study areas in S County and Y District had an excess mortality of 184/100,000 and 138.8/100,000, respectively. The mortality rates among people in the two control areas were lower than the average level (Table 3).

**Table 3:** Average excess mortality rates of cancers among people in study and control areas in S County and Y District compared to the rural national level (1/100,000), July 2002-June 2005

	Group	Total cancer	Esophageal cancer	Stomach cancer	Liver cancer	Colorectal cancer	Lung cancer
S County	Study area	184.0	57.5	28.0	28.6	4.0	49.2
	Control area	-61.9	-4.8	-23.4	-15.4	-2.9	-2.8
Y District	Study area	138.8	14.4	25.2	33.8	7.8	33.0
	Control area	-48.2	-3.4	-16.0	-15.4	-1.3	-8.5

## Discussion

In a location where there is no registration of COD and not all medical records are kept by family members after a death, it is very difficult to acquire accurate data on COD. VA can be used for this investigation, especially in places with a lack of health services and no vital registration of COD. In most previous studies on VA, VA was mainly used to assist COD diagnosis for infectious, infant, child, or maternal diseases. The usage of VA is increasing among adults in many developing countries, such as India [12], rural Ethiopia [13], and other African countries [14,15]. By means of VA, reliable COD can be inferred. VA can have high sensitivity for common diseases, and in particular for cancer. In India, the sensitivity of VA for cancer reaches 94% to 95%[16,17]. In China, its sensitivity reaches 85% and specificity exceeds 95% [4]. VA plays a significant important role in finding patterns of COD in these areas.

Accuracy is a major issue of concern for verbal autopsy. The accuracy of VA is influenced by many factors, such as COD, characteristics of the deceased, classification of COD, the design and content of the questionnaire, and procedures carried out in the field [18]. Several studies have attempted to assess the validity of the VA instrument by validating it against a "gold standard," namely medical records of people who have died [19]. However, in this study, we had few medical records, making it difficult to validate the results of VA. Therefore, in order to assess the COD from VA more accurately, strict quality control strategies were adopted. First, because deaths are commonly underreported as a result of cultural burial rituals and concerns in rural China [20], we collected death tolls from the registered permanent residence administration departments and countryside health departments to avoid underestimation. Second, we derived COD from verbal autopsy results using physicians' reviews. This approach is one of the most widely used in VA studies and is regarded as having high sensitivity and specificity for selected COD but low repeatability for deriving COD [19,21]. In this study, senior experts located in Beijing re-examined 5% of the deaths, selected by random sampling, after the local clinical experts completed the COD assignments. Third, to prevent reporting bias, we used VA to diagnose whether the deceased suffered from cancer. Because we lack a gold standard, we used the index of prevalence rates to test and verify the distribution of COD between the study and control areas in S County and Y District. The similarity in patterns of prevalence and mortality rates serve as an extra validation of the VA. The relationship of mortality and morbidity patterns between the study and control areas is useful to determine the accuracy of VA diagnoses and that the impact of the exposure tested (ie, water pollution) was being reliably measured by VA.

Based on the survey conducted from 1973 to 1975, both S County and Y District had low prevalence rates of cancer (around 70% of the national prevalence for rural areas). The 2005 investigation found that cancer prevalence rates remained low in control areas (60% to 65% of the 2000 national prevalence for rural areas), while prevalence rates increased dramatically in the study areas, where villagers relied on polluted water sources for drinking water. Prevalence rates were particularly high for gastrointestinal cancers. In this study, also we examined a number of risk factors, but we did not find significant differences between study and control areas. These risk factors included infections with *Helicobacter pylori *and hepatitis B, smoking, alcohol use, dietary behaviors (e.g., consuming pickled, smoked, or molded food), and indoor cooking and heating practices. Based on these, we can infer that there was a higher occurrence of cancer along the Huai River basin.

We made efforts not to impose biases during the VA process (for example, VA procedures were conducted similarly in the study and control areas). However, this study has limitations. First, retrospective study design poses risks for recall bias. A long recall period is likely to impair a respondent's ability to recollect and report relevant information. A recall period ranging from one to 12 months is generally thought to be acceptable. One validation study showed no significant effect on sensitivity or specificity using differences in recall period length of one to 21 months [19]. In this study, because all deaths were from July 1, 2002 to June 30, 2005, the recall period for respondents is longer. Therefore, we use the prevalence study to confirm the VA results. Second, the questionnaire used in this paper was long, and it took at least 30 minutes to complete. The VA questionnaire should be further improved in future studies to enhance its operability. Third, we used the 2000 DSP data as a reference to the general rural population, because 2002 to 2005 data were not available. Therefore, data from the study and control areas were not collected at the same time as the reference group (i.e., overall rural population). Finally, other cancer risk factors, such as other types of environmental pollution, might have confounded the observed patterns between the study and control areas. There may also be residual confounding from other unidentified cancer risk factors, which could affect the observed patterns.

## Conclusions

In conclusion, though limitations apply, the symptom-based VA can be used to infer COD in places where medical services are insufficient and people usually die at home. In rural China, where almost 80% of people die at home [22], using VA to assign COD can be useful and feasible.

Using VA, we have found evidence for higher cancer-caused mortality rates in the areas near the Huai River, where water pollution is a serious concern. Our findings demonstrate the feasibility of using VA as a diagnosis tool for cancer in rural China.

## Competing interests

The authors declare that they have no competing interests.

## Authors' contributions

XW participated in the design of the study, performed statistical analyses, and drafted the manuscript. MZ and ZT participated in the design of the study and performed statistical analyses. DD participated in drafting the manuscript. conceptualized the study, designed and organized of the study, and helped to draft the manuscript. All authors read and approved the final manuscript.

## Supplementary Material

Additional file 1 **Questionnaire (translated into English) used for verbal autopsy collection in this study**. Click here for file
