# Supplementary material for: Epidemiologic application of verbal autopsy to investigate the high occurrence of cancer along Huai River Basin, China
Source: Popul Health Metr. 2011 Aug 4;9:37. doi: 10.1186/1478-7954-9-37 (PMC3160930; doi:10.1186/1478-7954-9-37)
Supplement: Additional file 1 — Questionnaire (translated into English) used for verbal autopsy collection in this study. [file 1478-7954-9-37-S1.DOC]

**Questionnaire on Death Cause Inference**

**(Applicable to population aged 5 y/o and older)**

County

Town

Administrative Village

Natural Village

Respondent Name

Questionnaire Code

**Health Status Survey of Resident in**

**Focal Areas of Huai River Basin**

**Chinese Center for Disease Control and Prevention**

**Inform Consent**

Health status of people living along Huai River is an issue that the government pays great attention to currently. Chinese Center for Disease Control and Prevention has undertaken an investigation program on health status of residents along Huai River and launched field investigation from August to September 2005, in order to provide scientific evidence for intervention work to improve people’s health status.

As we are not able to do the general survey, only part of the people have been selected to attend our survey, and you are one of them. This survey is a questionnaire survey. We want to know something about the cause of death and related information of your family member(s) who passed away. The information collected from this survey will be kept in confidentiality, and only be used for related programs and scientific research.

You attendance is very important to this program. We are looking forward to your cooperation. If you yourself have already understood the information above, and are willing to attend this survey, please sign here. If you have any other question, please contact Dr. ________________, Tel: _____________________.

Thank you!

Respondent Signature: ___________________

Investigator_________________(Signature)

Date of Survey: ____(D) ____(M) ____(Y)

Supervisor_________________(Signature)

Date of Supervision: ____(D) ____(M) ____(Y)

**Part 1. Basic information of the Respondent**

1.01 Name of the respondent _____________

1.02 Telephone number of the respondent____________

1.03 Relationship of the respondent to the decedent

01-Father 02-Mother 03-Spouse 04-Sibling 05-Child

06-Other relative 07-Non-relative 08-Health worker 09-Not known

1.04 Did you live with the decedent before she/he died?

01-Yes 　02-No

**Part 2. Basic information of the Decedent**

2.01 Name of the Decedent_____________

2.02 Gender

　 01-Male　 02-Female 09-Not stated

2.03 Nationality

01-Han 02-Hui 03-Mongol

09-Others, please specify__________

2.04 Date of birth: ____(D) ____(M) ____(Y) (Solar calendar; plus 1 on month if lunar calendar)

2.05 Date of death: ____(D) ____(M) ____(Y) (Solar calendar; plus 1 on month if lunar calendar)

2.06 Place of death

01-Wards in hospital 02-Emergency room 03-Home or way to the hospital

04-Non-local or others______ 09-Not known

2.07 Marital status

01-Unmarried 02-Married 03-Widowed 04-Divorced

09-Not stated

2.08 Education

01-Graduate 02-Bachelor 03-Junior college 04-Secondary vocational school

05-Vestibule school 06-Senior high school 07-Junior high school

08-Primary school 09-Illiterate or semiliterate

2.09 Occupation (the longest occupation the decedent had)

01-Peasant 02-Worker 03-Teacher 04-Health worker

05-Civil Servant 06-Student 09-Others, please specify

**Part 3. The Death Situation Described by the Respondent** (Write strictly according to the statement of the respondent)

3.01 Please describe the disease or event which causes the death of the decedent in detail.

**Part 4. Survey on the Disease-related Symptoms and Conditions**

**“Execuse me, I want to ask you some questions about the disease symptoms which you consider may be related to the decedent’s death. Please answer patiently.”**

**Did she/he have the following symptoms after she/he suffered from the disease? (Please ask item by item)**

4.01 Did she/he have fever (have a high temperature)?

01-Yes 02-No 09-Not known

1. If YES, for how long? _______years

_______months

_______days

1. What was his/her fever like?

01-Persistent 02-Intermittent 09-Not known

1. Did she/he have afternoon fever?

01-Yes 02-No 09-Not known

4.02 Did she/he feel short of breath after light activity?

01-Yes 02-No 09-Not known

1. If YES, for how long? _______years

_______months

_______days

4.03 Did she/he feel short of breath when lying down, so that she/he couldn’t fall asleep?

01-Yes 02-No 09-Not known

A. If YES, for how long? _______years

_______months

_______days

4.04 Did she/he feel palpitation?

01-Yes 02-No 09-Not known

1. If YES, for how long? _______years

_______months

_______days

4.05 Did she/he have wheeze (expiratory dyspnea)?

01-Yes 02-No 09-Not known

A. If YES, for how long? _______years

_______months

_______days

1. What was his/her symptom like?

01-Persistent 02-Intermittent 09-Not known

4.06 Did she/he have cough?

01-Yes 02-No 09-Not known

A. If YES, for how long? _______years

_______months

_______days

B. Severity?

01-Mild 02-Modest 03-Severe 09-Not known

C. What was his/her cough like?

01-Dry cough 02-With white foamy sputum 03-With purulent sputum

04-With bloody sputum 05-Hemoptysis 06-Others________ 09-Not known

4.07 Did she/he have chest pain?

01-Yes 02-No 09-Not known

A. If YES, for how long? _______years

_______months

_______days

B. What was his/her symptom like?

01-Persistent 02-Intermittent 09-Not known

1. Was the chest pain located close to the sternum?

01-Yes 02-No 09-Not known

1. Did the chest pain have sudden onset or gradual progression?

01-Sudden onset 02-Gradual progression 09-Not known

4.08 Did she/he have diarrhea?

01-Yes 02-No 09-Not known

A. If YES, for how long? _______years

_______months

_______days

B. What was his/her symptom like?

01-Persistent 02-Intermittent 09-Not known

C. How many times per day? 　Times per day_______

4.09 What was his/her feces like when he/she was ill?

01-Yellow soft 02-Watery 03-Bloody purulent

04-Tarry 05-Others______ 09-Not known

**If you have chosen 02, 03, 04 or 05, please answer:**

A. For how long? _______years

_______months

_______days

B. What was his/her symptom like?

01-Persistent 02-Intermittent 09-Not known

4.10 Did she/he have stool with blood (fresh blood)?

01-Yes 02-No 09-Not known

A. If YES, for how long? _______years

_______months

_______days

B. What was his/her symptom like?

01-Persistent 02-Intermittent 09-Not known

4.11 Did she/he have bad appetite?

01-Yes 02-No 09-Not known

A. If YES, for how long? _______years

_______months

_______days

B. What was his/her symptom like?

01-Persistent 02-Intermittent 09-Not known

4.12 Did she/he mention retrosternal burning sensation or pain when swallowing?

01-Yes 02-No 09-Not known

A. If YES, for how long? _______years

_______months

_______days

B. What was his/her symptom like?

01-Persistent 02-Intermittent 09-Not known

4.13 Did she/he have dysphagia (difficulty in swallowing when eating)?

01-Yes 02-No 09-Not known

A. If YES, for how long? _______years

_______months

_______days

B. What was his/her symptom like?

01-Persistent 02-Intermittent 09-Not known

4.14 Did she/he have headache?

01-Yes 02-No 09-Not known

A. If YES, for how long? _______years

_______months

_______days

B. What was his/her symptom like?

01-Persistent 02-Intermittent 09-Not known

C. What was his/her headache like?

01-Dull or vague pain 02-Sharp pain 03-Swelling pain 09-Not known

D. Location of headache?

01-Whole head 02-Frontal 03-Occipital 04-Left or right half 09-Not known

4.15 Did she/he have hematuria (bloody urine)?

01-Yes 02-No 09-Not known

A. If YES, for how long? _______years

_______months

_______days

B. What was his/her symptom like?

01-Persistent 02-Intermittent 09-Not known

C. Was the hematuria symptom accompanied by pain?

01-Yes 02-No 09-Not known

4.16 Did she/he feel pain during urination?

01-Yes 02-No 09-Not known

A. If YES, for how long? _______years

_______months

_______days

B. What was his/her symptom like?

01-Persistent 02-Intermittent 09-Not known

4.17 Did she/he have symptoms of oliguria or anuria?

01-Yes 02-No 09-Not known

A. If YES, for how long? _______years

_______months

_______days

B. What was his/her symptom like?

01-Persistent 02-Intermittent 09-Not known

4.18 Did she/he urinate more frequent than normal every day?

01-Yes 02-No 09-Not known

A. If YES, for how long? _______years

_______months

_______days

B. What was his/her symptom like?

01-Persistent 02-Intermittent 09-Not known

4.19 Did she/he often feel thirsty, so that she/he would drink much water?

01-Yes 02-No 09-Not known

A. If YES, for how long? _______years

_______months

_______days

4.20 Did she/he feel stabbing pain on the feet?

01-Yes 02-No 09-Not known

A. If YES, for how long? _______years

_______months

_______days

B. What was his/her symptom like?

01-Persistent 02-Intermittent 09-Not known

4.21 Did she/he have abdominal (belly) pain?

01-Yes 02-No 09-Not known

A. If YES, for how long? _______years

_______months

_______days

B. What was his/her symptom like?

01-Persistent 02-Intermittent 09-Not known

C. Where was his/her abdominal pain located?

01-Upper abdomen ***If you’ve chosen 01, continue*** (01.1-Left upper abdomen 01.2-Right upper abdomen 01.3-Not known)

02-Lower abdomen ***If you’ve chosen 01, continue*** (02.1-Left lower abdomen 01.2-Right lower abdomen 01.3-Not known)

03-Periumbilical 09-Not known

4.22 Did his/her abdomen (belly) distend?

01-Yes 02-No 09-Not known

A. If YES, for how long? _______years

_______months

_______days

B. Did his/her abdominal distend with?

01-Sudden onset 02-Gradual progression 09-Not known

4.23 Did she/he vomit?

01-Yes 02-No 09-Not known

A. If YES, for how long? _______years

_______months

_______days

B. If YES, did the vomitus have putrefactive odor and overnight food in it?

01-Yes 02-No 09-Not known

C. If YES, was vomiting like jet?

01-Yes 02-No 09-Not known

4.24 Did she/he have hematemesis?

01-Yes 02-No 09-Not known

A. If YES, for how long? _______years

_______months

_______days

4.25 Did she/he have abdominal mass?

01-Yes 02-No 09-Not known

A. If YES, for how long? _______years

_______months

_______days

4.26 Did she/he have mental anomaly (personality and behavior changes, psychosis)?

01-Yes 02-No 09-Not known

A. If YES, for how long? _______years

_______months

_______days

4.27 Did she/he have loss of consciousness (coma)?

01-Yes 02-No 09-Not known

A. If YES, for how long? _______years

_______months

_______days

B. How did the loss of consciousness (coma) happen?

01-Suddenly 02-Gradually 09-Not known

4.28 Did she/he have hemiparalysis, i.e. paralysis (immobility) on one side of body?

01-Yes 02-No 09-Not known

A. If YES, for how long? _______years

_______months

_______days

4.29 Did she/he have paralysis (immobility) of bilateral lower limbs?

01-Yes 02-No 09-Not known

A. If YES, for how long? _______years

_______months

_______days

4.30 Did she/he have stiffness of the whole body?

01-Yes 02-No 09-Not known

A. If YES, for how long? _______years

_______months

_______days

4.31 Did she/he feel stiffness in the neck?

01-Yes 02-No 09-Not known

A. If YES, for how long? _______years

_______months

_______days

4.32 Did she/he have convulsion phenomenon (limb spasm, muscle twitching, convulsion, cramping not due to calcium deficiency or low temperture)?

01-Yes 02-No 09-Not known

A. If YES, for how long? _______years

_______months

_______days

B. When this symptom became worse, how many times per day? ________times

4.33 Did she/he have the phenomenon of weakened intelligence (becoming silly)?

01-Yes 02-No 09-Not known

A. If YES, for how long? _______years

_______months

_______days

B. Which type of the following?

01-Amnesia (unable to remember recent events and people)

02-Calculation decline (unable to do accounts)

03-Cognitive decline (unable to know the way or people)

04-Others 09-Not known

**---Female decedent aged 13 y/o and older---**

4.34 Did she have redness, swelling, or ulceration in her breasts?

01-Yes 02-No 09-Not known

A. If YES, for how long? _______years

_______months

_______days

4.35 Did she have lumps on the breasts?

01-Yes 02-No 09-Not known

A. If YES, for how long? _______years

_______months

_______days

4.36 Did she have vaginal bleeding, except for menstrual period?

01-Yes 02-No 09-Not known

A. If YES, for how long? _______years

_______months

_______days

B. Which of the following vaginal bleeding types?

01-Dripping during the intermenstrual period 02-Bleeding after menopause for many years 03-Bleeding after sexual behavior 09-Not known

C. Did the vaginal bleeding last until her death?

01-Yes 02-No 09-Not known

4.37 Did she have abnormal vaginal secretion (leukorrhagia, yellow, bloody, or with abnormal odor)?

01-Yes 02-No 09-Not known

A. If YES, for how long? _______years

_______months

_______days

4.38 Did she have vaginal mass, either felt by herself or discovered during gynecologic examination?

01-Yes 02-No 03-其他 09-Not known

**--- Female decedent aged 13-49 y/o ---**

4.39 Did she get pregnant during the last 1 year before she died?

01-Yes 02-No 09-Not known

If YES, how long was she pregnant? _______months

4.40 Did she give birth during the last 1 year before she died?

01-Yes 02-No ***(jump to 4.50)*** 09-Not known ***(jump to 4.50)***

If YES, how many days was it apart from her death? ______days

4.41 Was the bleeding severe when the labor pain started?

01-Yes 02-No 09-Not known

4.42 Was the bleeding severe during parturition (before the baby was expulsed)?

01-Yes 02-No 09-Not known

4.43 Did she have difficulty in delivering the fetus?

01-Yes 02-No 09-Not known

4.44 Did she have difficulty in delivering the placenta?

01-Yes 02-No 09-Not known

4.45 Did the parturition last too long (prolonged labor, the duration between rhythmic pain and baby expulsion was longer than 12 hours)?

01-Yes 02-No 09-Not known

4.46 Was it a Caesarean Section?

01-Yes 02-No 09-Not known

4.47 Was the obstetrical forceps or cephalotracter used in parturition?

01-Yes 02-No 09-Not known

4.48 Was the bleeding severe after baby expulsion?

01-Yes 02-No 09-Not known

4.49 How about the children’s health status?

01-Alive 02-Stillborn 03-Died within 7 days after delivery

　 04-Died after 7 days after delivery 05-Twins, one of whom died

4.50 Did she have abortion or miscarriage during the last 1 year before she died?

01-Yes 02-No 09-Not known

4.51 If YES, how many months/days before her death? _____mon____day

**-----Other symptoms (all decedents) -----**

4.52 Did she/he have swollen ankles?

01-Yes 02-No 09-Not known

A. If YES, for how long? _______years

_______months

_______days

4.53 Did she/he have joint swelling?

01-Yes 02-No 09-Not known

A. If YES, for how long? _______years

_______months

_______days

4.54 Did she/he lose weight?

01-Yes 02-No 09-Not known

A. If YES, for how long? _______years

_______months

_______days

B. What was the symptom like?

01-Rapid weight loss 02-Gradual weight loss 09-Not known

4.55 Did she/he look pale?

01-Yes 02-No 09-Not known

A. If YES, for how long? _______years

_______months

_______days

4.56 Did his/her lips look cyanotic?

01-Yes 02-No 09-Not known

4.57 Did his/her skin have abnormality?

01-Yes 02-No 09-Not known

A. If YES, for how long? _______years

_______months

_______days

B. Please describe the conditions of his/her skin?

01-Maculopapule 02-Herpes 03-Pustula

04-Subcutaneous hemorrhagic spots or petechia 05-Subcutaneous nodule

06-Phyma 07-Dermal ulcer 08 Others__ 09-Not known

4.58 Did she/he have facial swelling?

01-Yes 02-No 09-Not known

A. If YES, for how long? _______years

_______months

_______days

4.59 Did she/he have bilateral lower limb swelling?

01-Yes 02-No 09-Not known

A. If YES, for how long? _______years

_______months

_______days

4.60 Did she/he have swelling of the whole body?

01-Yes 02-No 09-Not known

A. If YES, for how long? _______years

_______months

_______days

4.61 Did his/her sclera (white of the eye) look yellow (jaundice)?

01-Yes 02-No 09-Not known

A. If YES, for how long? _______years

_______months

_______days

4.62 Did she/he have lumps on other regions of the body?

01-Yes 02-No 09-Not known

A. If YES, for how long? _______years

_______months

_______days

B. If YES, the location of the lump was

4.63 Did she/he have enlarged lymph nodes?

01-Yes 02-No 09-Not known

A. If YES, for how long? _______years

_______months

_______days

B. If YES, where was the location of the enlarged lymph nodes?

C. Estimated size? (Record as follows)

(Soybean-sized, peanut-sized, horsebean-sized, jujube-sized, apricot-sized, egg-sized)

D. Did the enlarged lymph node hurt?

01-Yes 02-No 09-Not known

E. Did the enlarged lymph node grow rapidly?

01-Yes 02-No 09-Not known

4.64 Did she/he have stuffy nose or rhinorrhagia (bloody nose)?

01-Yes 02-No 09-Not known

A. If YES, for how long? _______years

_______months

_______days

4.65 Did she/he have vocal hoarseness?

01-Yes 02-No 09-Not known

A. If YES, for how long? _______years

_______months

_______days

4.66 Did any injury (accident) happen to her/him before death?

01-Yes 02-No ***(jump to 5.01)*** 09-Not known ***(jump to 5.01)***

4.67 If YES, what kind of injury (accident) was it?

01-Car accident 02-Suicide 03-Animal or insect bite

04-Injured by others on purpose 05-Accidental poisoning (e.g., pesticides, alcohol)

06-Others_________ 09-Not known

4.68 What was the result of the injury (accident)?

01-Injured 02-Ill 03-Death 04-Not injured

09-Not known

A. If you’ve chosen 01, 02, or 03, for how long? _______years

_______months

_______days

**Part 5. The Healthcare Service Which the Decedent Received during Illness**

5.01 Did she/he see the doctor for this illness?

01-Yes 02-No ***(jump to 5.05)*** 09-Not known ***(jump to 5.05)***

5.02 If YES, have you kept the medical records, laboratory sheets, and other related materials in your family?

01-Yes 02-No 09-Not known

***If YES, please collect all the related materials. If original copies can’t be collected, please photocopy related materials, and put them in the questionnaire folder.***

5.03 If she/he saw the doctor, which level of hospital did she/he go to?

- 1. Village doctor:
  2. Township hospital: Department:
  3. County hospital: Department:
  4. Regional or city hospital: Department:

09-Not known

5.04 Did the doctor tell you about the cause of death of the decedent?

01-Yes 02-No 09-Not known

*If YES, what did she/he say?*

5.05 What medications did she/he take during this illness period? Can you recall some?

Expert Diagnosis Table

(Diagnose According to the Questionnaire on Death Cause Inference)

Questionnaire Code: Name of Decedent:

1. Could this case be diagnosed? 01 Yes 02 No

If NO, please specify reasons:

If YES, please fill in the following table:

| Diagnosis | Evidence for diagnosis (Please fill with question number in the questionnaire you refer to) | | | | | | | | Is the evidence sufficient^＊^ |
| --- | --- | --- | --- | --- | --- | --- | --- | --- | --- |
|  |  |  |  |  |  |  |  |  |  |
|  |  |  |  |  |  |  |  |  |  |
|  |  |  |  |  |  |  |  |  |  |
|  |  |  |  |  |  |  |  |  |  |

＊：01 Very sufficient 02 Sufficient 03 Not sufficient

| Diagnosis of the major disease causing death (please write the specific  disease name, not the symptoms or signs)  I. (**a**) Disease or condition causing death directly:____________________  (**b**) Disease or condition leading to disease (**a**):____________________  (**c**) Disease or condition leading to disease (**b**):____________________  II. Other diagnosis (other important conditions advancing but not causing death) | Estimated interval between onset and death  __________________  __________________  __________________ |
| --- | --- |

Signature of Expert: Date:

**The follow contents are filled by ICD coding experts organized by the program.**

Essential Cause of Death:

ICD-10 Code:

Statistics Classification ID

Signature of Expert: Date:

Expert Diagnosis Table

(Diagnose According to the Questionnaire on Death Cause Inference)

Questionnaire Code: Name of Decedent:

1. Could this case be diagnosed? 01 Yes 02 No

If NO, please specify reasons:

If YES, please fill in the following table:

| Diagnosis | Evidence for diagnosis (Please fill with question number in the questionnaire you refer to) | | | | | | | | Is the evidence sufficient^＊^ |
| --- | --- | --- | --- | --- | --- | --- | --- | --- | --- |
|  |  |  |  |  |  |  |  |  |  |
|  |  |  |  |  |  |  |  |  |  |
|  |  |  |  |  |  |  |  |  |  |
|  |  |  |  |  |  |  |  |  |  |

＊：01 Very sufficient 02 Sufficient 03 Not sufficient

| Diagnosis of the major disease causing death (please write the specific  disease term, while not the symptoms or signs)  I. (**a**) Disease or condition causing death directly:____________________  (**b**) Disease or condition leading to disease (**a**):____________________  (**c**) Disease or condition leading to disease (**b**):____________________  II. Other diagnosis (other important conditions advancing but not causing death) | Estimated interval between onset and death  __________________  __________________  __________________ |
| --- | --- |

Signature of Expert: Date:

**The follow contents are filled by ICD coding experts organized by the program.**

Essential Cause of Death:

ICD-10 Code:

Statistics Classification ID

Signature of Expert: Date:
